# Bayesian Inference for Generalized Linear Mixed Model Based on the Multivariate *t* Distribution in Population Pharmacokinetic Study

**DOI:** 10.1371/journal.pone.0058369

**Published:** 2013-03-08

**Authors:** Fang-Rong Yan, Yuan Huang, Jun-Lin Liu, Tao Lu, Jin-Guan Lin

**Affiliations:** 1 Department of Mathematics, Southeast University, Nanjing, China; 2 Department of Mathematics, China Pharmaceutical University, Nanjing, China; 3 State Key Laboratory of Natural Medicines, China Pharmaceutical University, Nanjing, China; 4 Laboratory of Molecular Design and Drug Discovery, China Pharmaceutical University, Nanjing, China; Sapienza University of Rome, Italy

## Abstract

This article provides a fully Bayesian approach for modeling of single-dose and complete pharmacokinetic data in a population pharmacokinetic (PK) model. To overcome the impact of outliers and the difficulty of computation, a generalized linear model is chosen with the hypothesis that the errors follow a multivariate Student *t* distribution which is a heavy-tailed distribution. The aim of this study is to investigate and implement the performance of the multivariate *t* distribution to analyze population pharmacokinetic data. Bayesian predictive inferences and the Metropolis-Hastings algorithm schemes are used to process the intractable posterior integration. The precision and accuracy of the proposed model are illustrated by the simulating data and a real example of theophylline data.

## Introduction

The population model is a pivotal element for the estimation of the individual pharmacokinetic parameters needed for dosage individualization. For a comprehensive discussion of the principles of population pharmacokinetic analyses see Ette and Williams *et* *al* [1]–[2]. One of the principal aim of population pharmacokinetic studies is to estimate the parameters associated with intra- and inter-individual variability in observed drug concentrations. Another important aim in population pharmacokinetic model is the establishment of relationships between parameters and covariates to explain parameter variability and facilitate dose adjustment decisions. So the explanation of the inter-individual variability in terms of subject-specific covariates is crucial for the study of population pharmacokinetics.

Many statistical models have been proposed to fit population PK (PPK) parameters. The most popular analytic statistic model for population PK data is the linear mixed model proposed by Laird and Ware [3]. In this model, the probability distributions for the response vectors of different individuals belong to a single family. However some random-effects parameters vary across individuals, with a distribution specified at the second stage. The first nonlinear mixed-effects modeling program introduced for the analysis of large amounts of pharmacokinetic data is NONMEM [4]. In the NONMEM program, inter- and intra-individual variability measures are combined, in a first-order approximation. Besides, many other more accurate methods have been incorporated in this software including Expanded Least Squares (ELS) and maximum likelihood method. In fact non-linear methods are used to estimate parameters of a chosen compartmental model. This method generally produces good results. For other nonlinear models for the analysis of population pharmacokinetic data see Wakefield *et al* [5]–[7], in which M-H algorithm is being used as an implementation of MCMC.

Ruth Salway *et al* [8] proposed a generalized linear model (GLM) with gamma distribution to deal with population PK data. But in a number of cases, especially during the development of new drugs, the structural pharmacokinetic model and inter and intra-subject variability models could be misspecified. This may lead to biased estimation for population parameters. Misspecification of 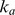 may lead to errors in the estimation of volume and PK parameters such as maximal concentration (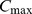). Graphical methods and Hosmer-Lemeshow type goodness-of-fit statistics can be used to detect misspecifications, as can be seen in Janet R [9] and Ivy Liu [10]. In the studies of other researchers, many semi-parametric and nonparametric methods have been proposed to reduce the bias of estimates [11]. However, in all of these methods mentioned above, at least one of the following important issues standout.

Firstly, all the models and methods have a common assumption that the residual error is normally distributed, but that assumption is not always proper. This assumption may lack the robustness against departures from normality and outliers and may also lead to misleading results. In the context of clinical trials with large numbers of observations on per subject, often one or two of them may give rather extreme response values, so a heavy tail distribution may be more appropriate than the (log) normal distribution. When we try to estimate the parameters of pharmacokinetic data with heavy tails, such (log) normality assumptions are inappropriate to outliers and it may affect the estimation of fixed effects and variance components seriously. Secondly, the associated intensive computation burden in the inference is a major challenge, and in some cases it can even be computationally infeasible. Particularly, for nonlinear longitudinal models the computational problem becomes much worse. Therefore, in our study, a multivariate *t* distribution for a generalized linear model is considered, which has two obvious advantages: (1) *t*-distribution is more robust for modeling data with heavy tails than the normal distribution, i.e. it is more prone to outliers. It approaches the normal distribution as ν (freedom degree) approaches infinity, and smaller values of ν yield heavy tails. (2) GLM model is easier to compute than nonlinear models. So in this paper, Bayesian method to estimate parameters in the GLM model is employed. The most pragmatic merit of Bayesian approach is the ability to take account of all parameter uncertainties. In aspect of Bayesian method research, lots of authors have advocated Markov Chain Monte Carlo (MCMC) schemes to deal with intractable posterior integrations, which can help reduce the difficulty of simulating directly from the posterior distribution. Wakefield AJ(1996) has proposed that MCMC methods used for hierarchical models [12]. O.Gimenez (2010) adopted a Bayesian framework with MCMC to carry out estimation and inference [13]. Chib considered several MCMC sampling schemes for hierarchical longitudinal models [14].

This article addresses these issues by modeling the response variable with outliers and using a Bayesian approach to investigate estimated parameters of generalized linear model proposed by Ruth Salway [8]. The rest of this article is organized as follows: “The Model” section describes the model and the chosen priors. The Metropolis-Hastings reject algorithm is constructed in the section of “Bayesian estimation and predictive inference”. “Simulation Study” section provides three simulations to illustrate the performance of our proposed method. Extensive model checking is carried out for the theophylline data in the section of “Application”. A conclusion is made in the part of “Discussion”.

## Methods

### Generalized Linear Model with *t* Errors Distribution

In the PPK study, usually one or two of the observations may give such a rather extreme response values that a heavy tail distribution may be more appropriate than the (log) normal distribution. Gomez et al. have introduced a multivariate generalization of the power exponential distribution which could effectively model heavy-tailed data [15]. This is a subfamily of the elliptically contoured distributions, including the multivariate normal distribution as a special case. Another better known subfamily is the multivariate Student *t* distribution. The strategic importance of these distributions arises because they only require simple modifications to the multivariate normal distribution which could be easily programmed.

The one-compartment model has been widely studied [16], [17]. The concrete expression is
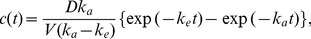
(1)Where c(t) is drug concentration which is a function of time, D is dose, V is the apparent distribution volume, and 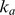 and 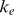 are respectively the absorption and elimination rate.

According to Ruth Salway *et al* [8], we can rewrite the one-compartment model (1) as
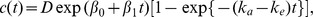
where 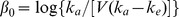 and 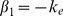. This model is really a transform of 1-compartment model. Let 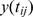 be the 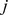 th 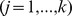 measured concentration for the 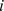
th 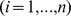 individual at time 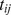. Then using a GLM and fitting the log-linear fractional polynomial model:



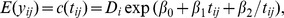
Here, 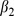 determines the absorption and we require 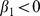 and 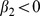 to ensure an increasing absorption phase and a decreasing elimination phase. Owing to the skewed nature of pharmacokinetic data, we shall use a log transform of the data. And for the GLM inference for other compartment model see Ruth Salway 2008 [8].

Let *t* Denote an 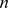-dimensional multivariate 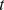 distribution with location vector 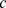, scatter matrix 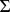 and degrees-of-freedom 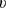, then a two-stage generalized linear random effect model that incorporates a finite mixture model could be rewritten as
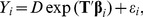
(2)where 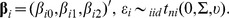


At the first stage represented by (2), 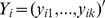 is the observation vector for the 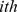 individual; 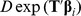 is the function defining the pharmacokinetic model, including subject-specific variable (e.g. dose). The design matrix 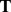 equals 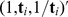, and 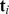 is the time schedule. 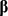 is the vector of fixed effects, representing the population parameters. 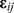 is the vector of random effects, and 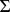 the positive definite covariance matrix. The distribution of 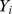 can be expressed as 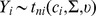 and interested parameters can be expressed in terms of 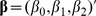, which representing the model parameters.

At the second stage.
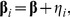
(3)where 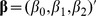 is the associated fixed effects, and 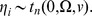


At the second stage given by (3), a finite mixture model is used to describe the population distribution, where 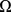 represents the correlation matrix. With equation (2) and (3), the population physiology parameters such as half-life period, peak concentration and time to peak (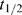, 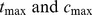) could be derived from 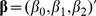 directly according to the formula given by Ruth Salway et al [8]

### Bayesian Inference

Methods such as Maximum likelihood techniques together with the algorithm such as EM, SAEM have been frequently used in GLMMs [18]–[19]. The computational burden is relatively small. Due mainly to recent advances in computing technology, the Bayesian sampling-based approach has been recognized as an alternative modeling strategy to offer the data-analysis. The ability to consider all parameter uncertainties is the merit in this approach. And MCMC techniques have revolutionized the field of Bayesian statistics by enabling posterior inference for arbitrarily complex models. Of course it is true that the methods are computer-intensive and compared to an “equivalent” maximum likelihood analysis, the overall run-time may be much longer. In our research, we adopt the Bayesian method for the model with the *t*-distribution, the same as that of Wakefield *et al* [12]. Based on the Bayesian theory, the posterior probability can be computed as follows:
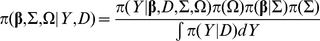
Since 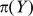 is a normalizing factor to ensure that 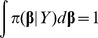, this factor in practice can be ignored unless alternative models are compared. Bayes theorem concerns the terms involving 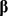 in essence and hence the complete posterior is often written as



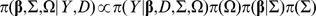



Here the posterior distribution is proportional to the product of the likelihood and the prior.

To complete a Bayesian formulation of the model above, one must specify a prior distribution for 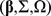. Suppose 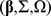 are independent priors, that is
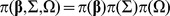



In this paper the traditional approach is adopted to avoid the confusion. In the absence of sufficient information of prior of 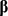, a popular method of avoiding improper posterior distributions is to use proper conjugate priors that are diffuse. For the reason that closed form posteriors or full conditionals could be derived analytically in this way, the distribution forms of priors are chosen traditionally for mathematical convenience. For example, an inverse-gamma prior is typically specified for 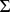 and 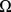 when the form of the normal density was assumed for each observation. Nowadays there exist several reliable methods for sampling from non-standard distributions, so it is worthwhile giving a little more thought towards one’s choice of priors. *First Bayes* (http://www.shef.ac.uk/stlao/lb.html) is an entry-level/educational Bayesian software package with a graphical interface, and it can be used to explore which distributions best reflect any prior information. For specific problems, prior uncertainty might be expressed better via a log-normal or uniform distribution. For example, Natrajan and Kass discussed the various options available for specifying non-informative priors for variance components [20]. In our study, the prior distributions are applied as follows:


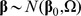
, and we assume 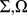 are diagonal matrix with 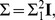 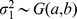, and 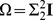, 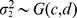. G(a,b) represents gamma distribution with scale parameter a and location parameter b. The single corresponding element of 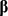 represents the population mean value, and the prior mean of which is typically set equal to an initial estimate. The element of 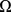 that corresponds to the gradients is usually set equal to zero, which represents none existence of covariate effects. Initial estimates based on previous studies may be a better choice, so in our study we use First Bayes or S-PLUS to test different values of c and d to find a pair which can result directly in a close match. Obviously the above discussion of priors for 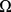 is also applicable to the 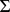 component. The hyper-parameters may also be chosen in a similar way.

Combining the complete-data likelihood function of model (2) with the prior distribution, we have the following joint posterior density of 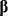.
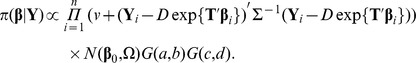



But, the posterior distribution is not tractable and thus we resort to the simulation-based methods. We provide Metropolis-Hastings algorithm schemes to deal with intractable posterior integration. The acceptance probability is calculated via a rejection algorithm. The implementation is described as follows:

The reject probability was denoted as
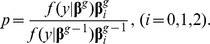



The rejection component proceeds are:

1. Generate alpha∼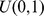, and 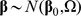, independently;
2. Accept 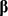 if alpha<min (1,*p*), 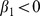 and 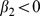, which are the constraints of the one compartment model, else keep the values of last iteration.

Then repeat step 1 and 2 until convergence, and the estimation could be obtained via analysis of the values of convergence part.

## Results

### Simulation Study

In order to illustrate that the model described above can be used for application, we develop three simulated examples.

#### Example 1

Firstly, we generated samples from normal distribution error terms, and analyzed these samples by normal error and *t*-distribution error in the M-H algorithm, respectively. The detailed processes are as follows.

The data containing 12 concentration measurements obtained from each individual over the successive 36 hours was simulated based on 10 subjects given a drug of 10mg dose each individual.

If 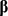 represent the collection of derived parameter vector 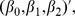 the population problem involves estimating 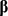 given the observed data 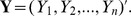 According to the model mentioned above, expressed by equation (2) and (3), we assigned that 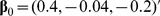. Set initial values of **β** originated from the initial estimates and then 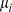 can be calculated. 10 subjects are simulated in Example 1. Time schedules of blood samples are set as [0.2 0.5 1 2 4 8 14 22 28 30 32 36]/h. The fixed effects are assumed to be normal, and the variance is 0.008. Random error is generated from the multivariate *normal* distribution with mean value equal to 0 and correlation matrix set as a diagonal matrix with element equal to 0.008. 100 samples have been obtained following the above sets.

Stable parameter estimators will be derived as long as there are enough iteration times through the following steps:

- Generate an identity matrix to save the posterior mean matrix, and calculate the initial mean values.
- Generate one random matrix sampled from the multivariate *t* distributions for error. Then the simulated observations equal the mean described in (2) plus the error matrix.
- Calculate the reject probability of the sample and judge the acceptance probability for each parameter 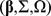 via the reject algorithm.
- Update the parameters, and do the iteration 5000 times. Iterating between step (ii) and step (iii) in the conceptual algorithm until convergence.
- Repeat the steps from (ii) to (iv) 100 times. In each repetition process, we discard these initial samples to reach steady state distribution. We estimated the necessary burn-in (e.g., 4000–5000 iterations) that collected from the posterior distribution. There are 100 samples in total. Therefore 100 parameter estimates are obtained, and then the means and 95% confidence intervals are computed from them.

**Figure 1:**
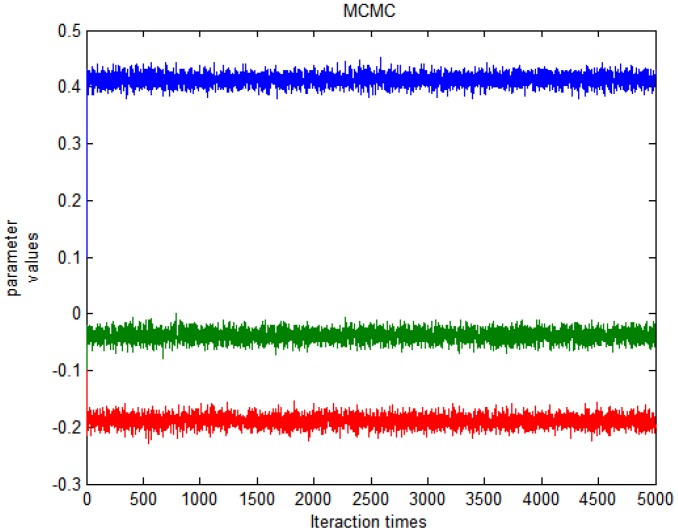
Examples of trace plots with MCMC.

**Figure 2:**
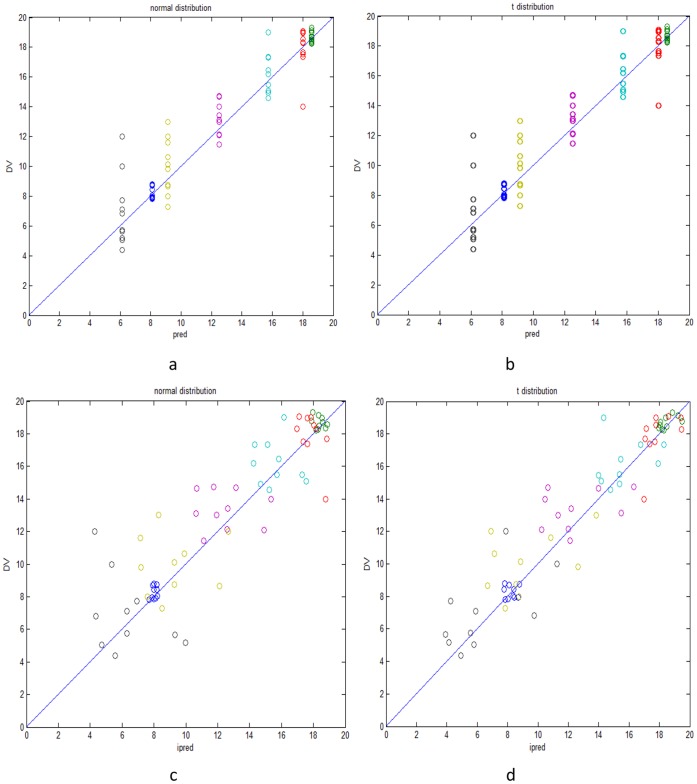
Relationship between population predicted value (pred, a, b) or individual predicted value (ipred, c, d) and observe values (DV) using normal distribution (a, c) and multivariate t distribution (b, d), respectively.

With the assumption, the generated data are typical concentration-time picture reflecting some unexpected conditions in the PK study. For the Bayesian models we use independent normal priors on the fixed effect. The markov chain as shown in Figure 1 could be obtained with the reject probability which is calculated by using the reject algorithm described before. For this problem, it is not easy to monitor convergence for 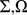. Even if incorrect initial valves are chosen, the chain will be rapidly converged to the previous specified values. Stable markov chain of the interested parameters is achieved after 5000 iteration times. The above process was implemented using Matlab software and the program codes are available in File S1. For example, Figure 1 shows that the posterior means ranges narrowly in the specified prior values. According to Figure 1, we can draw a conclusion that the convergence could be obtained rapidly with the initial values of (0.1, −0.1, −0.1). The means and 95% confidence intervals are computed from the last 1000 iterations. Point estimates and 95% interval confidence are presented in Table 1. For diagnostic plot to evaluate the goodness of fit see Figure 2 From table 1 and figure 2, it can be seen that the *t* distribution derives a close result with corresponding normal models.

**Table 1:** Estimate’s comparison between normal distribution (N) and *t* distribution (*t*).

parameters	Setting values	Estimated results (N)		Estimated results (*t*)
_β0_	0.4	0.3998(0.3997,0.4002)	0.4002(0.3994,0.4001)	
_β1_	−0.04	−0.0393(−0.0405, −0.0392)	−0.0400(−0.0401, −0.0398)	
_β2_	−0.2	−0.2001(−0.2004, −0.1996)	−0.1999(−0.2004, −0.1997)	
_σ1_	0.008	0.0092(0.0077,0.0105)	0.0088(0.0074,0.0104)	
_σ2_	0.008	0.0076(0.0072,0.0086)	0.0087(0.0076,0.0089)	

#### Example 2

**Figure 3:**
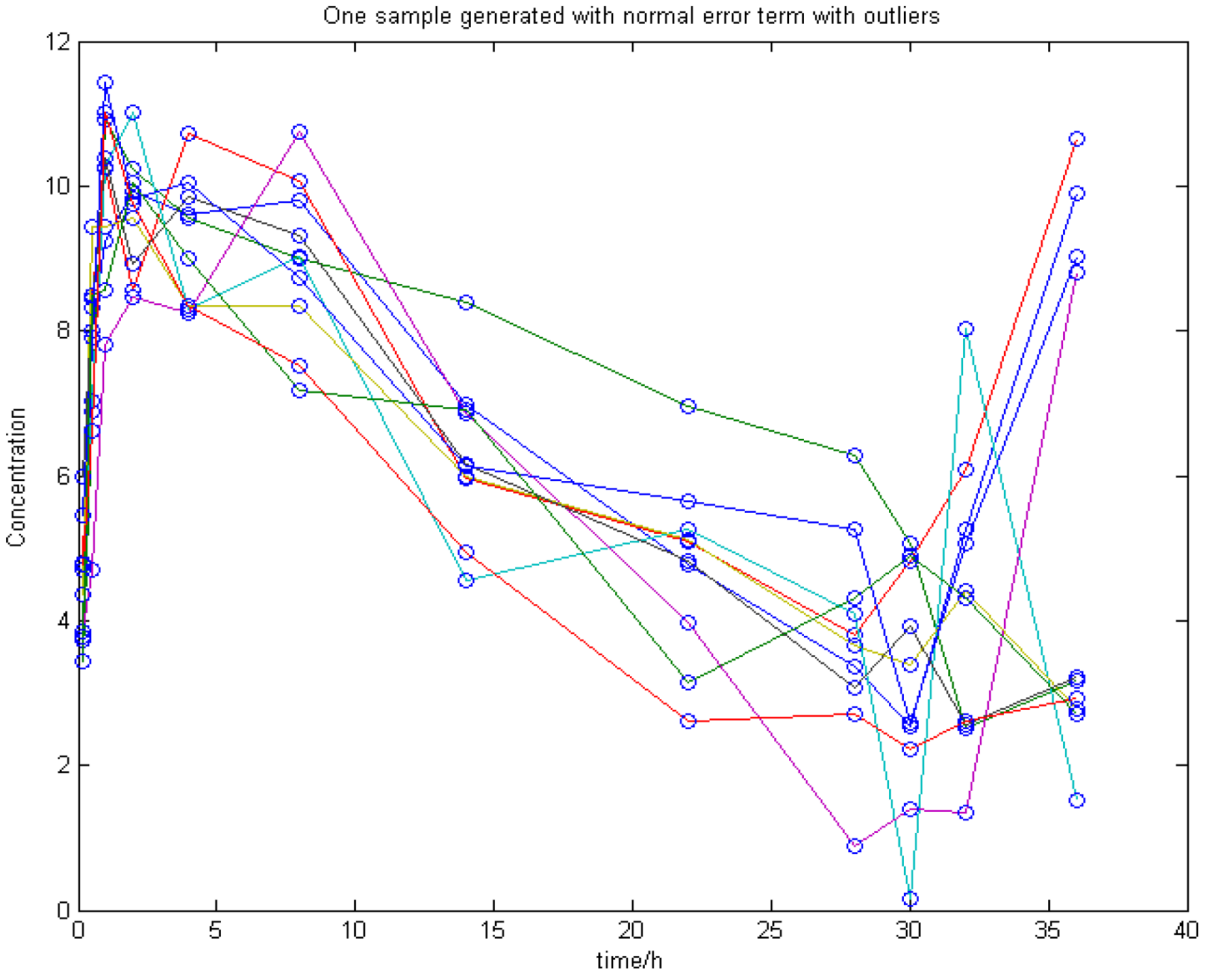
One sample of normal error terms with outliers.

The second simulation is to demonstrate that the *t* distribution approach yields better estimates of the population parameters when outliers are present. Unlike the above simulation, error terms of the sample are assumed to be normal distribution here. We random select 5% values of a sample, then plus a shift (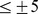) to these selected value as outliers. For a concrete example see Figure 3. Different from simulation in Example 1, we set 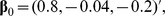 and 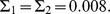 The results in table 2 shows that *t* distribution derives a more accurate point estimates and a relative smaller interval than normal distribution. The finding also shows that the generalized linear model with a *t-*distribution may achieve reliable results when the data exhibit outliers.

**Table 2:** Estimate’s comparison between normal distribution (N) and *t* distribution (*t*).

parameters	Setting values	Estimated results (N)		Estimated results (*t*)
_β0_	0.8	0.7997(0.7992,0.8001)	0.8000(0.7998,0.8001)	
_β1_	−0.04	−0.0401(−0.0405, −0.0398)	−0.0400(−0.0401, −0.0398)	
_β2_	−0.2	−0.2002(−0.2006, −0.1997)	−0.1999(−0.2001, −0.1997)	
_σ1_	0.008	0.0092(0.0076,0.0108)	0.0089(0.0074,0.0105)	
_σ2_	0.008	0.0077(0.0074,0.0081)	0.0084(0.0075,0.0090)	

#### Example 3

Unlike the above simulations, error terms of the sample are assumed to be *t* -distribution here. Again, we randomly select 5% values of a sample, then plus a shift (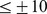) to these selected value as outliers. Different from simulation in example 2, we set 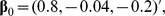 and 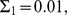 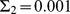. The results in table 3 show that *t* distribution derives a more accurate point estimates and a relative smaller interval than normal distribution. The finding also shows that the generalized linear model with a *t* distribution may achieve reliable results when the data exhibit outliers.

**Table 3:** Estimate’s comparison between normal distribution (N) and *t* distribution (*t*).

parameters	Setting values	Estimated results (N)		Estimated results (*t*)
_β0_	0.8000	0.9601(0.7065,1.1032)	0.7900(0.7812,0.8192)	
_β1_	−0.0400	−0.0021(−0.0490, −0.0011)	−0.0410(−0.0453, −0.0398)	
_β2_	−0.2000	−0.4093(−0.5978, −0.1927)	−0.1999(−0.2309, −0.1965)	
_σ1_	0.0100	0.0076(0.0052,0.0301)	0.0089(0.0071,0.0123)	
_σ2_	0.0010	0.0057(0.0003,0.0103)	0.0044(0.0007,0.0090)	

### Application

Here we present a true example. Real pharmacokinetic data are available from the resource Facility for Population Kinetics at http://www.rfpk.washington.edu. Initially this data set was used for Bayesian analysis of linear and non-linear population models by using the Gibbs sampling.

**Figure 4:**
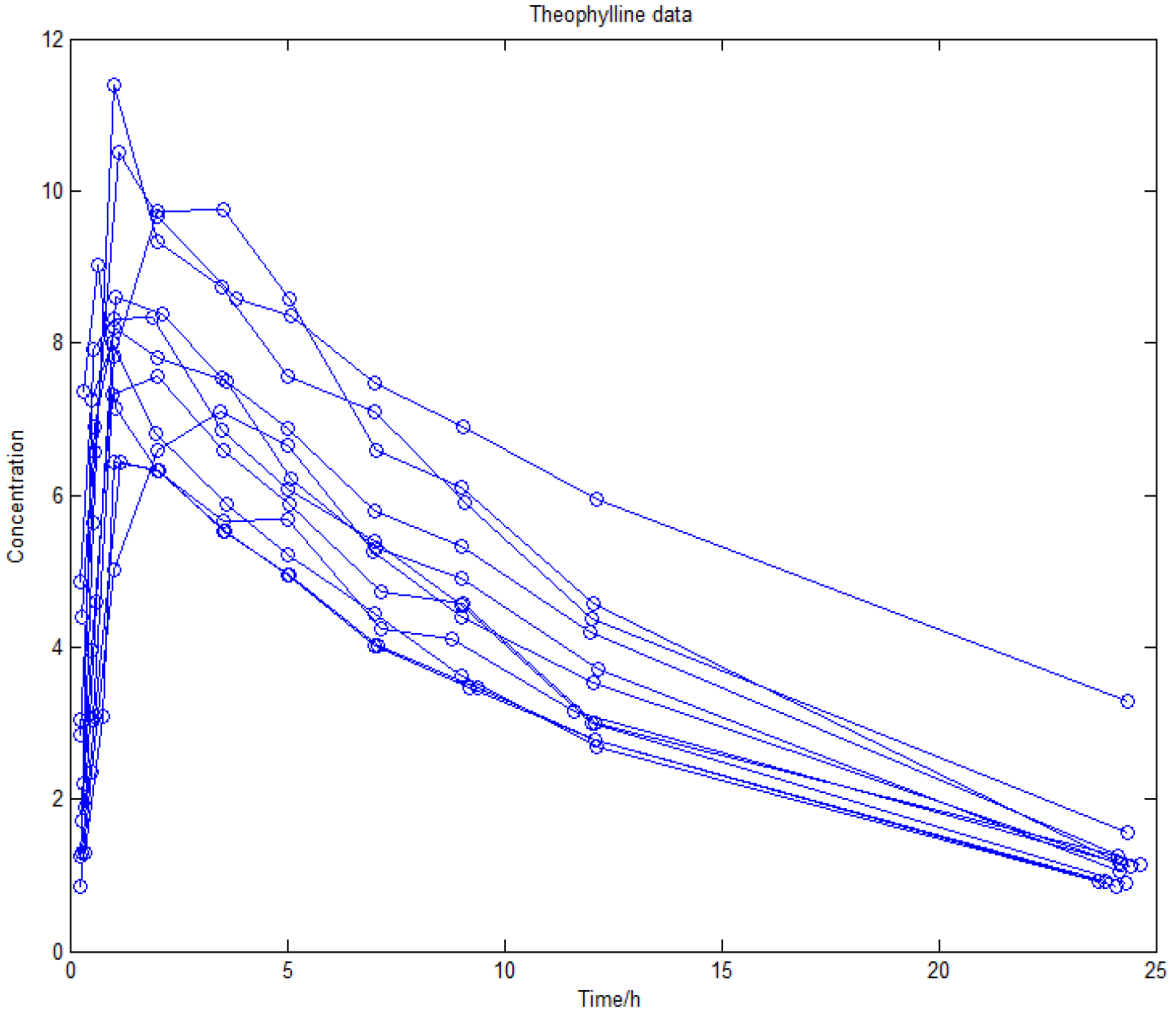
Observed concentrations of theophylline.

**Figure 5:**
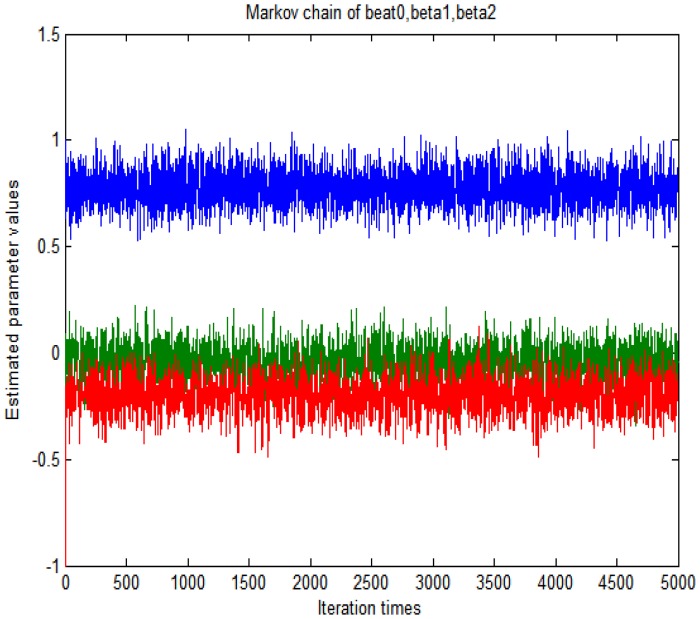
The markov chain of the parameters.

**Figure 6:**
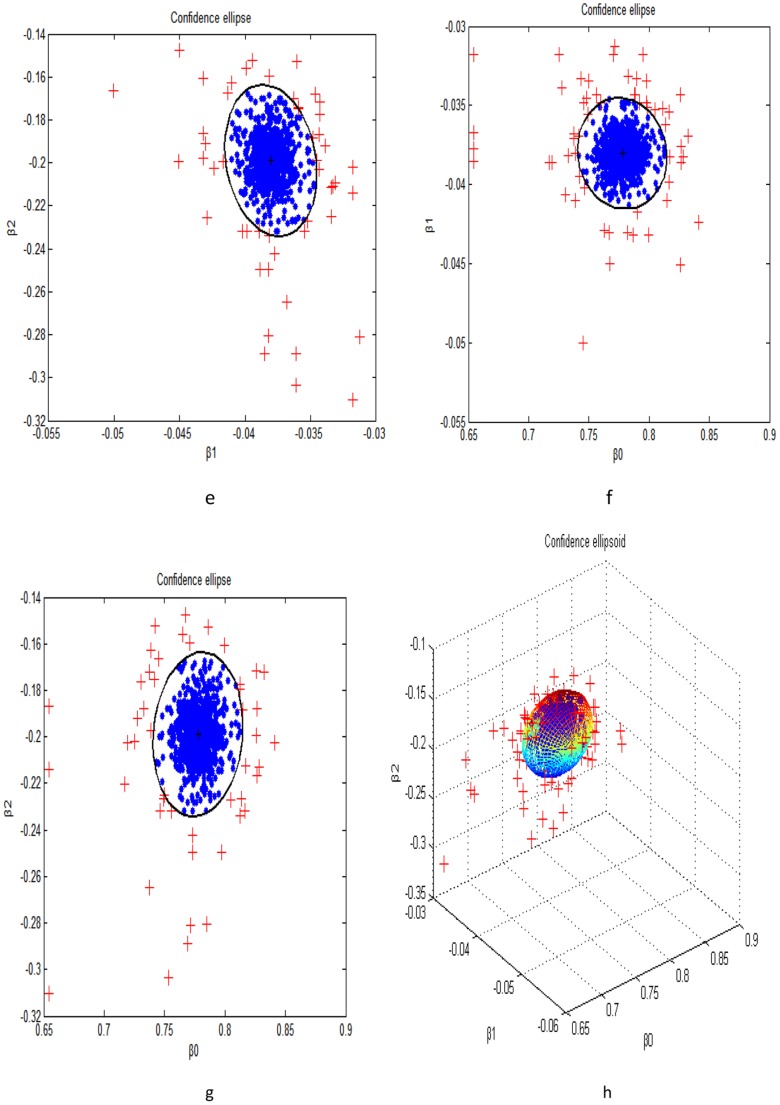
e,f,g: The confidence ellipse of the interested parameters. h: the confidence ellipsoid of the interested parameters.

For a description of the theophylline study sees Upton *et al* (1982). The parent drug concentrations in 24 hours are plotted in Figure 4. Here we apply our method to the data of 12 subjects given an oral dose of the antiasthmatic agent theophylline, with 10 concentration measurements obtained from each individual over the successive 25 hours. The data was originally analyzed in Upton *et al*. (1982) [21] and is available from the Resource Facility for Population Kinetics. The data is shown in Figure 4. It has been also used in Bayesian analysis of linear and non-linear population models employing Gibbs sampling with normal errors [22]. Wakefield suggested the data be analyzed by the generalized linear mixed model with the gamma error [8]. Now we apply *t*-distribution for generalized linear mixed model. Again, a convergence plot of markov chain has been obtained, as can be seen in Figure 5. Confidence ellipse and confidence ellipsoid pictures are shown in Figure 6. Most of the observations are located inside the domain. Ignore some exceptional values, this may suggest a good fit for the parameters. A comparable representation with the gamma distribution for the derived parameters can be seen in Table 4 and Table 5.

**Table 4:** Population model parameters in theophylline data.

parameters	Estimated mean	95% CI
_β0_	0.7778	(0.7768, 0.7787)
_β1_	−0.0379	(−0.0380, −0.0377)
_β2_	−0.1985	(−0.1993, −0.1978)

**Table 5:** Comparing PK parameter estimates for theophylline data.

	Gamma distribution	95% CI	*t* distribution	95% CI	Normal distribution	95% CI
*t_max_*	2.01	(1.68,2.38)	2.15	(2.05,2.24)	2.37	(2.20,2.53)
*c_max_*	8.82	(8.05,9.70)	9.46	(9.37,9.55)	9.28	(9.22,9.31)
*_t_*_1/2_	7.66	(6.99,8.42)	7.92	(7.85,7.99)	8.87	(8.64,9.10)

For performing the fitting of the model, we choose AIC information criterion to further analyze the results and asses the model. AIC information criterion, which can judge complexity of models, is a standard to evaluate the goodness-of -fit of models. After calculation, AIC values for the three models are obtained as 42.0077 for gamma model, 39.8176 for *t*-distribution model and 45.1087 for normal distribution model. The AIC value of *t*-distribution is smallest, so in this application it is proper to assume the error of pharmacokinetic model follows a *t*-distribution and it is better than normal distribution.

## Discussion

In population PK studies both random effects and within-subject errors are assumed to be normally distributed for mathematical convenience. However, such a normality assumption may be not suitable and in turn may affect the estimates of regression coefficients and variance components especially when the experimental data is thicker than normal tails or atypical observations. In this paper, we offered another distribution–multivariate *t-*distribution for Population PK data, and have obtained a more effective result than the Gamma distribution and normal distribution. In Example 1, results of *t*-distribution and normal distribution are close, indicating that *t*-distribution can be used when estimating the parameters in population pharmacokinetic models. In Example 2, PPK sample is produced from normal distribution and outliers are introduced. The results show *t*-distribution is more accurate than normal distribution for this sample. In Example 3, we introduced outliers from *t*-distribution, which still works better than normal distribution. We conclude that heavy-tailed *t* errors for generalized linear regression models are more stable than the normal error model, in the presence of outliers and it is an ideal method when processing clinical data.

We choose multivariate *t*-distribution for three reasons. Firstly, normal distribution is the limit of *t*-distribution. When the degree of freedom tends to be infinite, the *t*- distribution is exactly the normal distribution. If the degree of freedom is small, then *t*-distribution tends to be more suitable. Thus, for the real data, we should estimate the degree of freedom of *t*-distribution in the very beginning. In a Bayesian analysis of a model with *t*-distribution developed by Geweke (1999) [22], the degree-of-freedom, if unknown, must be sampled from its conditional distribution. In this paper, our approach is similar to the method described by Worsley and Friston (1995) and implemented in SPM99 [23]. This is a data driven method and independent of any specific preprocessing method. Obviously, if the number of effective degree of freedom is large enough (say, >50), approximate distributions can be used (e.g., normal or 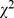). Secondly, *t*-distribution is advantageous over normal distribution especially when there exist outliers in the data because *t*-distribution is more stable than normal distribution. Thirdly, it only requires simple modifications to the multivariate normal distribution which could be easily programmed. It is obvious to use other distributions for extension.

Bayesian estimation is a good method for Population PK data (Wakefield *et al.* [24]). Wakefield [25] concluded that Bayesian inference was practically feasible for GLMMs, and provided an attractive choice to likelihood-based approaches. However there have been two major obstacles during its routine use: the specification of prior distribution and the evaluation of integrals which are required for inference. If the likelihood is correctly specified, the posterior distribution will be asymptotically normal with the mean of the true values, and variance covariance matrix may be given by the inverse of the expected information. Bayesian methods can be applied to the fractional polynomial GLMM, which can in turn be converted to PK parameters in straightforward fashion. In this study we placed priors on the model parameters (beta0, etc.) instead of derived parameters of interest, e.g., 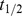, 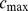 etc., to avoid complex calculation and inference. Though in the pharmacokinetic study, these derived parameters are more understandable and combine various sources of information.

In this paper we also discussed informal (graphical) methods, which could provide both informative diagnostic aids and easily-understood inferential summaries and were sufficient for practical purposes. Arguably the most useful graphical tool for assessing convergence is the “trace” plot, using such a plot an experienced analyst can usually detect when a single chain has reached steadily, i.e., at what point the samples become independent of the starting value. From Figure 1 and Figure 6, we could see that the simulation process have implemented convergence.

There are many problems that need further studies in the future. Firstly, in fact, missing data and censoring data are very common in population PK data analysis. So it is worthwhile to analyze missing data and censoring data by using *t* distribution. Secondly, in this paper, we provide the Bayesian method for fitting the pharmacokinetic data with *t*-distribution. Finding more efficient computing algorithm for population pharmacokinetic data with *t*-distribution should be considered in the future. Thirdly, choice of covariance structure should also be considered under the condition of *t*-distribution. Our methods are expected to expand to the context of variance structures including heteroscedasticity, autocorrelation structure, or even autoregressive time series structure.

## Supporting Information

File S1 **Simulation analysis matlab code.** Matlab code for performing the simulation analysis presented in the paper.
(DOC)
