# Supplementary material for: Bayesian Inference for Generalized Linear Mixed Model Based on the Multivariate t Distribution in Population Pharmacokinetic Study
Source: PLoS One. 2013 Mar 8;8(3):e58369. doi: 10.1371/journal.pone.0058369 (PMC3592804; doi:10.1371/journal.pone.0058369)
Supplement: File S1 — Simulation analysis matlab code. Matlab code for performing the simulation analysis presented in the paper. (DOC) [file pone.0058369.s001.doc]

Sample Code:

1.Illustation:

a. Main function: moni(), which is the main simulation function.

b.error generation function: randomerror();

c.Sample generating function: onesample(), which could generate a pharmacokinetic data with outliers.

d.M-H process: mh() and Nmh() which completes the parameter estimation for t and normal distribution, respectively.

2.Part of detailed code;

a. Main function and the parameter settings:

function mchain=moni()

%here needs the initial settings

for i=1:10

[itasigma,y]=onesample(sigma,beta0,beta1,beta2,t,D,itasigma);

mchainT=Nmh(y,sigm,bet0,bet1,bet2,beta0,beta1,beta2,itasigm,NUM,t,D); %%%normal distribution method

mchainN=mh(y,sigm,bet0,bet1,bet2,beta0,beta1,beta2,itasigm,NUM,t,D);

%%%t distribution method

postmeanT(i,:)=mean(mchainT');

postmeanN(i,:)=mean(mchainN');

end

b. %Randomerror function：generate random error matrix from multivariate t distribution;

function new=randomerror(sigma,n,ob)

if nargin~=3

error('please input sigma n observation number');

end

new=zeros(n,ob);

j=1;

for i=1:n

e1=mvtrnd(sigma*eye(ob),5,1);

new(i,:)=e1;

end;

c. %onesample function

function [itasigma,y]=onesample(sigma,beta0,beta1,beta2,t,D,itasigma)

sigma=0.01;beta0=0.413;beta1=-0.038;beta2=-0.198;D=10;,itasigma=0.0001

%;[itasigma,y]=onesample(sigma,beta0,beta1,beta2,t,D,itasigma);

if nargin~=7

error('please input sigma beta0 beta1 beta2 t n D itasigma');

end;

n=size(t,1);

ob=size(t,2);

e=randomerror(sigma,n,ob); %the random error matrix with obervations

[ita,y1]=miu(beta0,beta1,beta2,t,D,itasigma); % mean

y=y1+e; observation is the sum of this two values

%%%%%%%%%% Produce outliers: Choose 3 points randomly and add 2 to this point to make them outliers. If this part is excluded, there will no outliers.

site=floor(10+ (100-10).*rand(3,1));

y(site)=y(site)+2;

plot(t(1,:),y,'o-','MarkerEdgeColor','b');

%%%%%%%%calculate with normal distribution %%%%%%%

%Functioin Nmh：calculate the sample generated above with normal distribution：

d. %M-H

%ini=[sigma,beta0,beta1,beta2]; ini=[1,1,-1,-1,];

function mchain=Nmh(y,sigm,bet0,bet1,bet2,beta0,beta1,beta2,itasigm,NUM,t,D)

%if nargin~=7

% error('please input y,ini=sigma beta0 beta1 beta2 ita NUM t');

%end

n=size(y,1);

ob=size(t,2);

mchain=zeros(5,5000);

mchain(:,1)=[0.1 0.1 0.1 0.1 0.1];

postmean=zeros(5,1);

%miu1=zeros(n,ob);

for i=2:NUM

curr=mchain(:,i-1);

newsigma=unifrnd(0.0001,curr(1));

newe=Nrandomerror(newsigma,n,ob);

curre=Nrandomerror(curr(1),n,ob);

s=1;

c1= newsigma*eye(ob);%repmat(newsigma,1,ob);

c2=curr(1)*eye(ob);%repmat(curr(1),1,ob);

for j=1:n

s=mvnpdf(newe(j,:),0,c1)/mvnpdf(curre(j,:),0,c2)*s;

end;

fz=s*unifpdf(newsigma);

fm=unifpdf(curr(1));

mhratio=fz/fm;

alpha=min(1,mhratio);

if unifrnd(0,1)<alpha

curr(1)=newsigma;

else

curr(1)=curr(1);

end;

itasigm=unifrnd(0.0001,curr(5));

miu1=Nmiu(curr(2),curr(3),curr(4),t,D,itasigm);

miu2=Nmiu(curr(2),curr(3),curr(4),t,D,curr(5));

s=1;

for j=1:n

s=mvnpdf(y(j,:),miu1,c2)/mvnpdf(y(j,:),miu2,c2)*s;

end;

fz=s;

fm=1;

mhratio=fz/fm;

alpha=min(1,mhratio);

if unifrnd(0,1)<alpha

curr(5)=itasigm;

else

curr(5)=curr(5);

end;

newbeta0=normrnd(beta0,curr(5));

miu1=Nmiu(newbeta0,curr(3),curr(4),t,D,curr(5));

miu2=Nmiu(curr(2),curr(3),curr(4),t,D,curr(5));

s=1;

for j=1:n

s=mvnpdf(y(j,:),miu1,c2)/mvnpdf(y(j,:),miu2,c2)*s;

end;

fz=s*normpdf(bet0);

fm=normpdf(curr(2));

mhratio=fz/fm;

alpha=min(1,mhratio);

if unifrnd(0,1)<alpha

curr(2)=newbeta0;

else

curr(2)=curr(2);

end;

newbeta1=normrnd(beta1,curr(5));

miu1=Nmiu(curr(2),newbeta1,curr(4),t,D,curr(5));

miu2=Nmiu(curr(2),curr(3),curr(4),t,D,curr(5));

s=1;

for j=1:n

s=mvnpdf(y(j,:),miu1,c2)/mvnpdf(y(j,:),miu2,c2)*s;

end;

fz=s*normpdf(bet1);

fm=normpdf(curr(3));

mhratio=fz/fm;

alpha=min(1,mhratio);

if unifrnd(0,1)<alpha

curr(3)=newbeta1;

else

curr(3)=curr(3);

end;

newbeta2=normrnd(beta2,curr(5));

miu1=Nmiu(curr(2),curr(3),newbeta2,t,D,curr(5));

miu2=Nmiu(curr(2),curr(3),curr(4),t,D,curr(5));

s=1;

for j=1:n

s=mvnpdf(y(j,:),miu1,c2)/mvnpdf(y(j,:),miu2,c2)*s;

end;

fz=s*normpdf(bet2);

fm=normpdf(curr(4));

mhratio=fz/fm;

alpha=min(1,mhratio);

if unifrnd(0,1)<alpha

curr(4)=newbeta2;

else

curr(4)=curr(4);

end;

mchain(:,i)=curr;

end;

%%%%%%%%mean function： calcultion of posteriori mean to get the parameter estimates.

postmean=mean(mchain(:,NUM-1000:NUM)')';

end;

%%%calculate with t distribution %%%%%%%%%%%%%%%%%%%%%%%%

%M-H

function mchain=mh(y,sigm,bet0,bet1,bet2,beta0,beta1,beta2,itasigm,NUM,t,D)

if nargin~=7

error('please input y,ini=sigma beta0 beta1 beta2 ita NUM t');

end

n=size(y,1);

ob=size(t,2);

mchain=zeros(5,5000);

mchain(:,1)=[sigm bet0 bet1 bet2 itasigm];

postmean=zeros(5,1);

%miu1=zeros(n,ob);

for i=2:NUM

curr=mchain(:,i-1);

newsigma=unifrnd(0,curr(1));

newe=randomerror(newsigma,n,ob);

curre=randomerror(curr(1),n,ob);

s=1;

c1=newsigma*eye(ob);

c2=curr(1)*eye(ob);

for j=1:n

s=mvtpdf(newe(j,:),c1,3)/mvtpdf(curre(j,:),c2,3)*s;

end;

fz=s*unifpdf(newsigma);

fm=unifpdf(curr(1));

mhratio=fz/fm;

alpha=min(1,mhratio);

if unifrnd(0,1)<alpha

curr(1)=newsigma;

else

curr(1)=curr(1);

end;

c=curr(1)*eye(ob);

newbeta0=normrnd(curr(2),curr(5));

miu1=miu(newbeta0,curr(3),curr(4),t,D,curr(5));

x1=y-miu1;

miu2=miu(curr(2),curr(3),curr(4),t,D,curr(5));

x2=y-miu2;

s=1;

for j=1:n

s=mvtpdf(x1(j,:),c,3)/mvtpdf(x2(j,:),c,3)*s;

end;

fz=s*normpdf(bet0);

fm=normpdf(curr(2));

mhratio=fz/fm;

alpha=min(1,mhratio);

if unifrnd(0,1)<alpha

curr(2)=newbeta0;

else

curr(2)=curr(2);

end;

mchain(:,i)=curr;

end;

end;
